# The Twenty-Eighth Annual Meeting of the Texas State Medical Association

**Published:** 1896-05

**Authors:** 


### The Twenty-Eighth Annual Meeting of the Texas State Medical Association.

In pursuance of announcement, the Texas State Medical Association met in Fort Worth, on the 28th of April, ult., and held a four days' session. The programme, as published in the last issue of the JOURNAL, was carried out; and altogether it was an interesting, and perhaps profitable meeting. Scientifically considered, it may be said to have been a success; in as much as a large number, and some really excellent papers, were read. One, of more than ordinary importance, that of Ex-Superintendent White, State Lunatic Asylum, was read. It advocated State aid for the epileptics, in accordance with the suggestions of the TEXAS MEDICAL JOURNAL of April. It elicited much interest, and a committee was appointed to memorialize the Legislature on the subject of colonizing epileptics. The committee appointed by the President consists of Drs. F. S. White, Terrell; A. N. Denton, Austin—both ex-superintendents of the State Lunatic Asylums; Dr. E. D. Capps, of Fort Worth; Dr. A. M. Douglass, of Covington, an Ex-State Senator, and Dr. H. L. Tate, of Lindale, Smith county.

Dr. White's paper appears in full in this issue.

There were many other valuable papers read,—too many to particularize;—and in speaking of this one we do not wish to be understood as discriminating, or as being biased.

. . . . . . .

Moreover, the occasion was honored by the presence of some distinguished visitors from abroad. Dr. Peterson, who has made much fame by founding, or securing the aid to found the Craig Colony of Epileptics in New York, and whose paper on the subject we referred to in our last issue, was present, and read a paper, as per announcement; also Prof. T. L. Crofford, the well known gynecologist of Memphis, and the universally known and popular Dr. J. M. Kellar, of Hot Springs, were there. Dr. Stoddard, of Pueblo, Colorado, was a visitor too, and had a good paper on "Some Mistakes in Gynecology." (The writer of this suggested to Dr. Stoddard, operating on a gravid uterus by mistake for fibroid, for instance,—a recent occurrence in St. Louis.) Dr. Stoddard's paper and Dr. Peterson's will both be published in the TEXAS MEDICAL JOURNAL soon.

. . . . . .

Numerically considered, the meeting was hardly up to the average, and far below expectations. It will be remembered that at last meeting attention was pointedly called by the President, in his message, to the "decline and fall off" of the Association, and a committee was appointed to investigate the causes, and to report,—and also to suggest means of remedying it. This committee, it will be remembered, consisted of Drs. Wooten and others, and at said last meeting they made a preliminary report, suggesting that the cost of membership be reduced,—cost of Transactions ditto,—papers limited to twenty minutes, and some other suggestions not now recalled. Thereupon a committee—consisting of Drs. T. J. Wilson, of Sherman, chairman; T. D. Wooten, Austin; J. F. Y. Paine, Galveston; M. D. Knox, Hillsbury, and Bacon Saunders, of Fort Worth—was appointed (at Dallas meeting, last year) to further consider the subject, and to suggest a mode of action best calculated to arrest the falling off of membership, promote cohesion and harmony; and to further consider and report upon medical legislation, and a whole lot of other matters, either one of which would have occupied the time of an ordinary committee. The committee did an extensive correspondence during the interval between the Dallas meeting and the one just held, enlisting the aid of physicians in all parts of the State, and there was an energetic whoop'em up all along the lines, with the intention of making a grand rally at Fort Worth, and starting on a new road to progress and success from this on. Under these circumstances, and considering also the personal popularity of the President and of the committee, on whose account more than usual interest was taken in the matter, it is a little disappointing to have to record that there were not exceeding one hundred and fifty present, all told, and rarely as many as one hundred in the hall at any one time.

. . . . . .

But what was lacking in numbers was made up in enthusiasm. Sixty new members were added to the roll,—the largest accession at any one time the Association has ever had. It remains to be seen how many will be dropped for non-payment of dues. In the recent past, the latter has out numbered the new members; hence the "decline and fall off." It is to be hoped now, that the tide has turned, and that new members will be added every meeting without a corresponding or greater number being dropped. For—

Certain changes were made, in accordance with the recommendations of the committee above mentioned, which it is hoped, will permanently remedy the evil complained of, and that the Association will rapidly grow in numbers and influence beyond its palmiest days of the past.

. . . . . .

Chairman Wilson, for the committee, made a most exhaustive report, an evening session (the first day) being held for the hearing. Prominent amongst the recommendations were important changes in the constitution and by-laws. The proposed changes were read and acted on *seriatim*, and consist of the following: The nominating committee, heretofore consisting of one member from each county represented, is now composed of one representative from each congressional district, thirteen in all. All the delegates from each district hold a caucus, and elect a spokesman or representative, whom they instruct or not for president. This nominating committee selects two names, and submits them to the convention, to be balloted for. (In the present instance, Drs. J. C. Loggins, of Ennis, and Bacon Saunders, of Fort Worth, were selected, and on ballot Dr. Loggins was elected.) This proposition met with much opposition, but was finally adopted. It was held by some to be dangerous to entrust the election of president to thirteen men; and by the bye, in this connection a recommendation was made which was the occasion of much feeling and some excitement. The committee, it seems, in a resolution preliminary to the subject of medical legislation, stated in effect, that in the past "questionable methods" had been resorted to by gentlemen to secure their election, and it was proposed to obviate all such in the future by this resolution. It brought out a vigorous protest from President Coleman, who took occasion to say, that if it was intended for him the cap did not fit. Others who had been president were hurt, and wanted it understood that they did not take the insinuation to themselves. It was a bomb-shell. Harmony prevailed to an alarming extent to the point where this came in. Old members were dumbfounded. That a committee, composed of one who had himself been president—two or three others of whom were regarded as prospective presidents, should have made such an insinuation—an impeachment, indeed, of all ex-presidents, was thought to be as unfortunate and indiscreet as it was astonishing. Dr. Coleman's remarks appear below—at the same time he expressed himself on other points:

"GENTLEMEN—I wish to call your attention to a fact, that throughout the discussion of this paper has not been touched upon. Dr. Wilson is here, and if the other members of that committee are here, I hope they will hear every word I say. I would, for no cause, do anything to reflect upon them, but I wish to enter a protest, a vigorous protest, against the allusion to the measures which have been used, so far as seeking to obtain office is concerned. Gentlemen, what is the natural conclusion? That I, that Dr. Becton, that Dr. Sam R. Burroughs, and that other gentlemen who have been honored with the presidency of this Association have resorted to such measures. Gentlemen, I do not mean to say that they did accuse us of it, but that when it goes out to the world that that paper was read before this Association, it is the natural inference that such measures have been used to obtain these offices. I want to say, that as I expect to enter into judgment for every thought, and for every word, and for every deed; if my record was as clear on all matters as it is on that, I will have a great deal better record than I expect to have. Never have I spoken, never have I solicited any gentleman to support me for that position. It came to me unsolicited, and, of course, I esteem it the greatest honor of my life. Not one word did I put upon record; I never wrote any letters, or in any way solicited support directly or indirectly. I thank you for your indulgence in this connection, in allowing me to say these few words."

. . . . . . .

Other changes of the constitution and by-laws were adopted, all of which went into immediate effect. I don't know by what kind of manœuver or rule of procedure they got over the provision that "any proposed change of constitution shall lie over a year." They were as follows: The initiation or membership fee ($5) is abolished; annual dues $5, constitutes the sole expenses to new and old members alike. It was pointed out that only new members get any benefit from this, as the yearly dues remains $5, there is no relief to old members—nor inducement added—for them to keep up their membership.

The Vice-Presidents are elected by the nominating committee of thirteen, and constitute a committee—each year to appoint all the section officers, "to give them something to do," as Secretary West urged, they being "only ornamental appendages."

The three Vice-Presidents chosen are Dr. A. N. Denton, Austin; J. S. Letcher, Dallas; David Cerna, Galveston, in the order named.

. . . . . .

☐ The Omnibus Committee—through Chairman Wilson—also, under the head of needed medical legislation, reported "a bill to regulate the practice of medicine," which the committee had agreed on as being the thing needed. It was read at the night session, but could not be voted on by sections on account of the lateness of the hour. It was brought up next morning and raised Cain for "a couple or two of hours." It proposed a board composed of six physicians, *four homeopaths* and *two* eclectics. The other provisions were after the stereotyped order. Meantime the Smith County Medical Society had prepared a bill which was read by Dr. Woldert, not as a substitute, but for comparison, "that the best features of each might be incorporated into one bill." But as both provided for a mixed board, and there were no essential differences, Dr. Woldert's bill was finally lost sight of, and all the discussion turned on the committee bill. There was firing all along the line, heaviest in the center, where the homeopaths and eclectics were entrenched, as it was claimed, behind the State constitution—that "no preference shall be given any school of medicine"—a heresy, a bugaboo—that served to whip into line even some of the vaunted "old guard." The old guard surrendered—but under protest—and one sole survivor still kicked,—the editor of the Red Back. The gentlemen "took their medicine" with the meekness of the proverbial lamb. Dr. Becton made a protest, but finally "acquiesced in the will of the majority."

Dr. Daniel said: "I am unalterably opposed to recognizing homeopathy in the manner contemplated by this bill, or in any other manner. I have opposed it by tongue and pen—in season and out of season, all my life; and although I find myself almost alone in this fight, I will resist it till my expiring breath, and if vanquished I will die with my boots on. Why, gentlemen, in asking for a board with homeopaths and eclectics represented, we stultify ourselves, and compromise our self-respect and honor. It is a complete surrender to a handful of pretenders. Every effort at medical legislation on the part of this Association year after year has been defeated by the misrepresentations and machinations of this class; no one denies it. We have not been able to secure medical legislation against their wishes, without *their consent*, and now do you propose to go to them, with hat in hand and say humbly: 'Gentlemen, we crave your kind permission to have a bill passed; upon what terms may we hope to succeed?' You offer them representation far out of proportion to numbers, in the hope that that will buy their consent. How do you know that they will not want more? Why, sirs, think of it. When I had the honor to serve this Association on its Legislative Committee—and I served several years—there was a homeopathic delegation at the Capitol also, and they had published a card, saying: 'Our medicines are harmless; they won't kill anybody; why can't we be left alone?' We went to them and said: 'You say your medicines are harmless and will not kill, and you want to be left out of consideration. We will insert a clause exempting from examination and from prosecution *every homeopath who will agree to practice homeopathy solely*, and adhere to your medicines, which, you say, are "harmless." Will *this* satisfy you and disarm your opposition?' 'No,' they replied, 'we reserve the right to practice as we please!' Gentlemen, is this not quackery, pure and simple? And fraud besides? To pose as homeopaths—to pretend to practice on the rule of *similars* and to give 'harmless' medicines—and at the same time to attempt to practice *medicine* by a system of which they know absolutely nothing, and which is fundamentally opposite to that which they claim to practice. Is this not quackery? And you now voluntarily offer to form an alliance with them to secure an enactment to exclude *all other* quacks! There is no difference in quacks, except in name and degree, and as the ends of the desired legislation is the suppression of quackery, it should embrace all quacks. Patrick Henry exclaimed: 'Is peace so sweet or life so dear as to be purchased at the price of chains and slavery? Forbid it, Almighty God!' In the language of Patrick Henry (altered to suit the occasion)

I ask, is medical legislation so essential—of such vital importance to this association—as to be purchased at the price of professional pride and honor, and personal self-respect? Forbid it, Almighty God. I know not what you and others think of (not 'this life,' as P. H. said, but of this bill) but as for my single self, I will not *take the soup*, under any circnmstances, and will resist to the last, every attempt at its forcible administration—after the manner in the story. No homeopathy in mine. I hope this clause will be stricken out."

It was gratifying, later, to see our President, who, although in the chair, with his hands tied by parliamentary usuages, overstep the bounds fixed by custom, and enter his solemn protest also. Here is what he said:

"In regard to the portion of the report relating to the board of medical examiners, I insist that the words homeopathic and eclectic should go out of the motion as it prevailed. That we, educated physicians, should frame a bill regulating the practice of our profession, and then leave it to the legislature to do whatever they think best with it. Here is the point I want to make. I do believe gentlemen, it will never do, in justice to ourselves, who are members of the State Medical Association, to let it go out that we have put our indorsement on a bill which recognizes these men on the outside.

"I thank you, gentlemen, for your very courteous attention. I thank you for it, and allow me to repeat it, that it is with the utmost respect. I know it is out of the usual line for such a speech to be made at this time."

. . . . . . .

It was finally agreed to strike out the offensive clause. Coleman was the Blucher—who turned the tide of this Waterloo; and the single champion who stood fighting all alone may be likened somewhat to the boy on the burning deck, and will be, by some, no doubt, thought to be just as big a fool. But, consistency is a jewel, rare these days, and for my part, I would rather let all the quacks have an equal chance, than collogue with one set to keep out the other.

. . . . . . .

The yearly performance of appointing a committing to go to Austin to lobby for this bill- -minus the homeopathy—was gone through with, and Drs. Denton, McLaughlin, Loggins, and Blailock were appointed.

. . . . . . .

The disposal of the medical bill about wound up the work mapped out by the omnibus committee except as to the publication of the Transactions. The Association has been squealing for several years at the excessive outlay for a volume of Transactions; that of last year exhausting the funds and incurring a debt of $150, and the committee proposed to try the plan of what they called "journalizing the Transactions;" thought it could be done cheaper. Somebody had an axe to grind, but it didn't grind worth mentioning, and, that somebody offered to publish the Transactions in that somebody's journal for a dollar a year. But, the matter having been left to another committee—Drs. Denton, White, Cunningham and Blailock, the majority reported in favor of continuing the present form of publication; i. e., in one volume, but insisted on its being gotten out for less money. (It stands to reason that if the Secretary could have gotten it done for less he would have done so, and it is hard to understand how he is to obey instructions unless he gets cheaper printing and cheaper paper). Dr. Denton made a minority report, favoring the publication in a journal; not in a journal of the Association, because they all must know that it takes something more than a few hundred subscriptions to run a journal, but to let it out by contract; and one bid of a *dollar a year* was already on file. The majority report was adopted; and after an immense amount of talk the matter remains *in statu quo*. Old members pointed out that the experiment of journalizing had been tried in the eighties, when the Association numbered twice as many as now, and Dr. Wilkinson found that he couldn't do it (in the then *Medical Record*), at $2, and threw up the job.

. . . . . . .

In the interest of retrenchment, the Association cut the $150 salary of the faithful old Treasurer in half. This is his twenty-second year of consecutive service, and cut off the pay of the publishing committee (Secretary West, ex-officio chairman), but leaves the Secretary's salary, proper, at $300.

. . . . . . .

The Transactions was gotten out in 1885–6–7–8–9 and '90, in satisfactory style and at satisfactory price; there was never any complaint. But then, the work was done by Dr. Daniel, at Austin, and by a non-union printer, Von Boeckmann, the same who prints the *Red Back*, and union printers at Galveston, it is said, will not bid against him; and so, he was excluded, after

1890, when the Secretary resigned, and the present incumbent took the work of getting out the book.

. . . . . . .

THE PAUPER ACT.—Away at night, at 11 o'clock on the night set to consider the Omnibus Report, after the changes in the by-laws had been made, and the balance of the report left over for next day, Dr. J. W. McLaughlin, of Austin, sprung his pet scheme, in the shape of a resolution, about to this effect and in these words, in part: "Resolved, that this Association memorialize the Texas legislature to *transfer* the State Quarantine to the United States Government."!!!!!!!!! (Mr. President, will you please *transfer* my household expenses and the expense of providing for my family, to somebody else? That's about a parallel.)

We hardly know why the author of the resolution took that time and occasion to introduce his resolution. It was on the night of the first day—a special session—the only night session,—and set aside for a specific purpose—and at the close of the business (hearing Omnibus Report); there were hardly twenty-five members in the room. Why was the doctor in such haste to get it off? A matter of such vast importance, it seems to me, should have been brought before the Convention when there was a full house—in the day time—in order that it might be discussed. It was railroaded through in ten minutes, at about 11:30 p. m., when nearly everybody had gone away, and there was but one there to oppose it. It was actually *adopted* by the few present, Dr. Daniel alone voting against it. He called attention to the fact that the resolution asks the legislature to do an impossible thing,—*transfer* the cost of quarantine of the State of Texas, to the United States Government. The legislature has no such authority. The legislature might petition Congress to pass an act for the relief of Texas, on the plea, should it be made, that Texas is too poor, too mean, or too ignorant, or lazy, to enforce efficient quarantine; but there is no reason to believe that Congress would do even this. Dr. Daniel assured them, that in submitting such request, the most preposterous and utterly absurd he ever heard of, emanating from so respectable a source, this Association put itself in a position to surely be refused and just as surely to be laughed at. He offered an amendment to the effect that the legislature, at the same time, "*transfer*" to the general government the border defense against Indians and raiders, and the interior police also, and disband our Texas militia and our Ranger guard; but the amendment was lost.

. . . . . . .

As stated, this remarkable resolution was adopted. It was championed by some of the best men present,—notably Drs. Loggins (now president), W. R. Blailock, J. H. Sears, M. D. Knox, and others, and these gentlemen, I believe, with Dr. McLaughlin, constitute the committee who are to present the petition. In course of the discussion. Dr. Knox said, in effect, that in adopting this resolution we were aligning ourselves with other States. Dr. Daniel asked him to name a State that had taken the initiative or had adopted that course. He said "New York." Dr. Daniel asked: "Do you mean to say that New York has, by her legislature, or otherwise, asked the National Government to relieve the State of the duty and expense of quarantine? "No," he said, "but the Marine Hospital Service had a quarantine in New York." Dr. Knox could, with the same propriety, have named Georgia, or Alabama, or even Texas herself; for at every coast station on the Atlantic and the Gulf, the Marine Hospital Service has an officer on duty; but it does not, in the least, interfere with the administration of State quarantine. The law distinctly states, that in case a State quarantine is, in the opinion of the Supervising Surgeon-General, Marine Hospital Service, for any reason "inefficient," it can be strengthened by the United States Marine Hospital Service, but it can not be otherwise interfered with. And here we wish to recall, for the information of the gentlemen who advocate this step, the fact, that the only time in the history of the Quarantine Service, when the Marine Hospital Service undertook to "strengthen" a State quarantine, was at Brunswick, Ga.; United States Marine Hospital Service officers displaced the State officers, and in consequence of inexperience on the part of the young man they put on duty, and his want of knowledge of yellow fever, he contracted the disease (after it had been effectually suppressed at quarantine), and carried it into the city and lighted up an epidemic; and—died!

. . . . . . .

A State Board of Health.—Dr. McLaughlin and his colaborers in the matter of quarantine transfer, propose simultaneously with the proposed easing off of the load of quarantine from the shoulders of the Texas government to saddle her with the expense of a board of health. The doctor is not satisfied with the successful and economical administration of what he and others call a "one-man quarantine;" and insists that a board of health will do better and be cheaper. It is difficult to see how better results could be obtained under any system,—for the State has been, for years, notoriously free from infectious disease; and where a case of small-pox or other infectious disease has appeared, it has been promptly squelched, and no epidemic of any magnitude has resulted. And the expense of all local outbreaks has fallen, and by law do fall upon the county where the disease occurs.

Now, with the Gulf and border quarantine "transferred" to Uncle Sam's shoulders, what will there be left for a State Board of Health to do, that is not now done—better,—by county physicians in each county, and *without cost to the State* at all? Is it proposed to transfer the administration of internal sanitation back from the several counties to the general government? Before the present law went into effect that was the rule; the State government paid for local epidemics—and after paying thousands of dollars for such things, there remain, to my certain knowledge, seventy odd thousand dollars of unpaid bills inci dental to small-pox management by a former State Health Officer. Do these gentlemen propose to go back to that method, and have a board of health to take this work off the hands of county physicians (who are subordinate to the State Health Officer in matter of infectious disease), and saddle the expense again on the State government? If not—what will there be for a board to do?

. . . . . . .

A State Board of Health is a popular idea; but I am persuaded the profession do not fully understand the situation. Under operation of existing methods the entire cost to the State for quarantine is very light. Dr. McLaughlin in his midnight resolution recited that the cost of maintaining quarantine for the four years, 1891–4 (Hogg's administration), had been $210-,000. Now Dr. McLaughlin kuew, or if not, should have known, that of this amount, over $21,000, had been especially appropriated to build and equip the new station at Galveston, and a fumigator at Sabine Pass, and, in 1892, there was a cholera scare which added to the expense. If the doctor wished to be just, and to convey an accurate idea of the expense of quarantine to the State government, he should have taken the cost of last year. When Governor Culberson went into office he saw the necessity for retrenchment and advised it; he asked the State Health Officer for an estimate of the expenses of his department. The legislature having reduced the pay of all quarantine officers—the State Health officer made an estimate of $33,000. And this amount has been amply sufficient for all ordinary purposes, and covers the entire expense to the State of protection against infectious disease, with a margin of over $2000 unexpended. Dr. McLaughlin must have known this, and a spirit of fairness, it strikes us, should have prompted him to state these facts.

. . . . . .

Referring to the discussion of the homeopathic clause in the bill, we were not altogether alone in the fight as stated. There was considerable opposition felt, but little expressed, and excepting Dr. J. T. Harrington, we do not now recall any one who spoke against it besides Dr. Becton and myself, and Dr. Becton —as stated—finally "caved." There were one or two others who opposed it in a few remarks. The hall reverbrated so with the least sound that hearing—ten feet away—was almost impossible. The clause was finally stricken out—after having been adopted by an overwhelmning majority.

. . . . . .

The election of Dr. J. C. Loggins, of Ennis, is a well deserved compliment,—a fitting recognition of his fidelity to the Association, and his fearless discharge of every duty imposed upon him; and they have have been many, and onerous and unpleasant. He has worked his way up from the humblest private in the ranks. He has been chairman of section, member of various committees, and for four years served on the Judicial Council, and that, too, during the stormiest times, when clear sight and fearless courage were requisite to steer clear of breakers, and prevent the foundering of the ship. She would have gone to pieces at Waco, in 1890, but for a level-headed Judicial Council composed of such men as Loggins, Isaac E. Clark, A. C. Walker, R. B. Gardner, Bev. West, Sam Burroughs and others. These men bore the brunt of the storm, and have stood the cursing of the disgruntled,—the storm which cleared the atmosphere and left all serene with smiling skies. And now Loggins is President. I wish all those named above could be President, and if there is anything better than President, I wish they could be that.

. . . . . .

Paris, Texas, the village of the extreme North, was selected as the next place of meeting. A woeful mistake. Too far from Center and South and East and West; not sufficient hotels, and members don't like to be quartered on the town. It is to be hoped—and the JOURNAL urges it now with all earnestness—that the place of meeting be changed, at once; if not, there will be a failure in point of numbers in attendance. Some central point, Waco, for instance, should be the permanent place of meeting, or Dallas or Fort Worth.

. . . . . .

The time of meeting every year, the last week in April, conflicts with the closing exercises of the Texas Medical College, and this prevents the attendance of those members who are connected with the school. At this meeting, only Secretary West (Professor) and Professor Cerna were there. Professors Paine, Smith, Randall, Clopton, Thompson, Keiller, and Morris, were missed.

. . . . . .

An elegant ball and banquet was given at Hotel Worth, in honor of the convention. Drs. Field and Saunders, and their ladies, gave the delegates a lovely reception.

. . . . . .

The familiar face of Dr. Sam R. Burroughs was missed from the meeting,—first time in twenty years; ditto our old friend Powell; ditto many others of the old standbys.

. . . . . .

The Treasurer, Dr. Larendon, Reported $365.25 on hand. The Secretary, Dr. West, reported as follows:

| | | |
|---|---|---|
| Members | | 355 |
| Honorary members | | 24 |
| New members at Dallas | 36 | |
| Honorary | 3— | 39 |
| | | 418 |
| Dropped | 31 | |
| Died | 5— | 36 |
| | | 382 |

Leaving membership, all told, active, 355; honorary, 29; just one hundred more than we had in 1882.

. . . . . .

The hall used by the Convention, the new $400,000 court house, was an unfortunate selection. The "acoustics" was not considered in building it, and one couldn't hear himself think. It roared like the Austin dam.

. . . . . .

The proposition submitted by the omnibus committee to have homeopaths and eclectics on the board of medical examiners, was supported by many good men; but we are satisfied, after talking with many, that they are under the impression that a the law says "no preference shall be given any school of medicine," if we ask for a straight board we will be refused. That is so, no doubt, but in the name of consistency, let us not ask for a mixed board. If the legislature shall refuse to grant any other kind of board and force this on the profession, why, we can not help it; but there is much difference in accepting what they will give and asking for it. Should the regular profession grant this recognition—voluntarily—it opens the way to consultation and full fellowship with this element.

. . . . . .

A graceful episode of the meeting was the presentation to the Association, by President Coleman, of a gavel made from the wood of a tree growing on the grave of McDowell. Dr. Steele Bailey, Secretary of the Kentucky State Medical Asssociation, procured and forwarded the wood from which the gavel was made, and a vote of thanks wus accorded him.

. . . . . .

A resolution was offered by Dr. J. T. Wilson, that since Dr. J. O. Williams, of William Penn, Washington county, Texas, claims the honor of having performed the first three symphiseotomies ever performed in the United States, the president appoint a committee of three to investigate the truth of the statement, that, if true, Texas might claim the honor before the world. Adopted.

. . . . . .

The following new members were admitted: Drs. J. H. Jenkins, Caldwell; L. D. Peeples, Navasota; J. P. Wier, J. W. Comfort, Woodbury; S. L. Terrell, Dallas; E. M. Williamson, George's Creek; E. J. McKinney, DeKalb; B. G. Prestidge, Alvarado; J. R. Nichols, Greenville; W. E. Menefee, Cleburne; C. C. Walker, Gainesville; Jno. S. Turner, Granbury; J. H. McCracken, Mineral Wells; Robt. F. LaMonde, Denver, Col.; G. R. Taber, Bryan; R. L. Miller, Decatur; J. W. McFall, Lipan; S. G. Bittuck, Ringgold; A. O. Scarborough, Snyder; J. J. Eargle, Proctor; Jas. H. Smart, Dallas; T. J. Dodson, Bartlett; E. E. Norvell, Bynum; W. F. Chambers, Austin; W. B. Anderson, Meridian; J. A. Cozby, Azle; W. R. Sedberry, Clifton; H. C. Gilbert, Smithfield; S. J. Froshang, Norse; R. E. Myers, Kemp; W. A. Wood, Hubbard City; W. T. Baird, Dallas; B. W. D. Hill, Dawson; and W. G. Kimbrough, Robt. G. Davenport, W. M. Yandle, Joseph Alexander aud D. N. Shropshire, whose postoffice addresses were not stated; J. L. Kennedy, Galveston; T. S. Booth, Ardmore, I. T.; Jos. A. Mullen, Houston; T. N. Self, Joshua; W. T. Starley, Jr:, E. B. Blalock, Woodlawn; I. H. Hunt, Big Springs; J. E. Dodson, Vernon; Ernest McMahon, Marshall; C. M. Yater, Grandview; G. T. LaGrande, Graham; E. A. Woldert, Tyler; T. F. Smith, Mexia; D. S. Reed, Wortham, and Vard H. Hulen, Galveston.

Prof. Crofford, of Memphis, was made an honorary member.

